# Reasons for individual and concurrent use of vaped nicotine and cannabis: their similarities, differences, and association with product use

**DOI:** 10.1186/s42238-021-00097-7

**Published:** 2021-08-27

**Authors:** Danielle M. Smith, Lynn Kozlowski, Richard J. O’Connor, Andrew Hyland, R. Lorraine Collins

**Affiliations:** 1 Department of Community Health and Health Behavior, School of Public Health and Health Professions, University at Buffalo, State University of New York, Buffalo, NY 14214 USA; 2 Department of Health Behavior, Division of Cancer Prevention and Population Sciences, Roswell Park Comprehensive Cancer Center, Elm & Carlton Streets, Buffalo, NY 14263 USA

**Keywords:** Nicotine, Cannabis, Co-use, Vaping, Reasons for use, Motivations for use

## Abstract

**Background:**

Understanding similarities, differences, and associations between reasons people vape nicotine and cannabis may be important for identifying underlying contributors to their co-use.

**Methods:**

A cross-sectional survey of 112 co-users of vaped nicotine and cannabis was conducted in 2020. A convenience sample of participants was recruited for the survey using Amazon Mechanical Turk. Participants responded to questions about their reasons for individual nicotine and cannabis product use and co-use and rated their level of agreement using numerical scales. Mean ratings for each reason for use subscale were examined across all participants and compared using paired samples *t* tests. Associations between reasons for use ratings and product consumption behaviors were examined using linear and logistic regression analyses.

**Results:**

Cannabis vaping and smoking exhibited similar mean ratings for user experience and product/substance-related reasons for use. Mean ratings for reasons related to product utility were similar for cannabis vaping and nicotine vaping. Mean ratings for utility-related reasons for use were higher for cannabis vaping than cannabis smoking (mean (SD), 3.6 (± 1.0) vs. 2.6 (± 1.2), *p* < 0.0001). On average, harm reduction-related reasons for use were rated higher for nicotine vaping than cannabis vaping (2.4 (± 1.6) vs. 1.8 (± 1.4), *p* < 0.0001). Regression models showed higher average ratings for utility-related (*b* = 0.32; 95% CI, 0.03-0.60) and harm reduction-related (*b* = 0.21; 95% CI, 0.04-0.37) reasons for nicotine vaping were associated with more frequent nicotine vaping (both *p* < 0.05). Higher average ratings for instrumentality-related reasons for co-use corresponded with more frequent monthly nicotine vaping (*b* = 0.26; 95% CI, 0.08-0.44) and higher odds of ever chasing cannabis with nicotine (aOR, 3.06; 95% CI, 1.29-7.30).

**Conclusions:**

Vaping serves purposes that differ by substance; nicotine vaping was more closely related to reducing tobacco smoking-related harms, and cannabis vaping was more closely related to circumventing social problems posed by cannabis smoking. Lifetime sequential co-use practices and more frequent nicotine vaping were associated with enhancing the intoxicating effects of cannabis. While replication of these findings using non convenience-based sampling approaches is warranted, results underscore the need to consider shared and unique aspects of nicotine and cannabis vaping, as well as cross-substance interactions between nicotine and cannabis.

**Supplementary Information:**

The online version contains supplementary material available at 10.1186/s42238-021-00097-7.

## Introduction

Approximately 20% of U.S. tobacco users, aged 12 years and older, engage in concurrent use (co-use) of nicotine and cannabis (U.S. Department of Health and Human Services, Substance Abuse and Mental Health Services Administration, Center for Behavioral Health Statistics and Quality, 2021). While the term refers to individuals who use both substances, co-use also encompasses specific practices, such as sequential use (i.e., immediately following use of cannabis with nicotine, or “chasing”), and co-administration (i.e., using nicotine and cannabis in the same delivery mechanism, such as blunts or spliffs) (Peters et al., 2012; Peters et al., 2014). Co-use of smoked tobacco and cannabis has been linked to increased risk of dependence on nicotine and cannabis, more severe experiences of respiratory illness, and increased exposure to smoke-related toxicants linked to the development of diseases later in life (Peters et al., 2012; Peters et al., 2014; Agrawal et al., 2012; Brook et al., 2010; Meier & Hatsukami, 2016; Smith et al., 2020). Vaping is continuing to take hold as a new mode of delivery for both nicotine and cannabis with observed increases in current nicotine vaping among U.S. young adults from 5.4-6.9% over the period from 2014-2018 (Bandi et al., 2021), and increases in past month cannabis vaping from 5-14%, and 8-17% among U.S. college and non-college young adults, respectively (National Institute on Drug Abuse, 2020). These increases in vaping have presented a number of important questions in studying co-use.

Understanding the factors that influence whether people vape nicotine or cannabis are important, as they are often linked to corresponding patterns of product use that can directly affect user health (including use frequency and quantity). Many users of nicotine and cannabis (in both smoked and vaped forms) report similar reasons for using for both substances, including enjoyment, peer influences, experimentation, boredom, and relaxation (Lee et al., 2007; Lee et al., 2016; Saddleson et al., 2016). Those who report vaping these substances also express similar reasons for use related to mode, with vaping being perceived as a healthier, cleaner form of drug delivery for both nicotine and cannabis (Saddleson et al., 2016; Aston et al., 2019). However, there are differences in reasons for vaping that vary by substance. Among those who use nicotine-containing e-cigarettes, 72% cite actively trying to cut down or quit tobacco cigarette smoking as a key reason for vaping (NAtional Academies of Science, Engineering, and Medicine, 2018; Coleman et al., 2017). Conversely, those who vape cannabis report doing so to heighten its subjective effects, and for the convenience and discretion afforded by vaping cannabis compared to using the drug through smoked means (Lee et al., 2016). The population of individuals who use both nicotine and cannabis significantly overlaps, with approximately half of all cannabis users reporting use of nicotine-containing products, and one in five tobacco users reporting use of cannabis (U.S. Department of Health and Human Services, Substance Abuse and Mental Health Services Administration, Center for Behavioral Health Statistics and Quality, 2021; Krishnasamy et al., 2020; Smith & Goniewicz, 2020). Currently, similarities and differences between reasons for vaping nicotine and cannabis among these dual users, and similarities and differences in reasons for vaping or smoking cannabis, have not been explored.

In addition, co-use behaviors involving sequential use and co-administration of nicotine and cannabis are practiced to enhance the subjective effects of cannabis. Yet, little is known about differences in engaging in these practices or related reasons for co-use in the context of vaping. Berg et al. (2018) developed a scale to measure reasons for smoked tobacco and cannabis co-use among young adults, which assessed dimensions of use related to enhancement of intoxication, product substitution, social context, and experimentation (Berg et al., 2018). However, we are not aware of any studies that have applied this measure elsewhere, including populations other than young adults. The degree to which these components of co-use apply to populations other than young adults is unclear, as is their possible link to frequency of nicotine and cannabis vaping, sequential co-use practices, and product co-administration.

The current study provided data on reasons for using vaped nicotine, vaped cannabis, smoked cannabis, and co-use of both substances. We sought to address the following questions: (1) What are the main reasons co-users of vaped nicotine and cannabis choose to use these products, both individually and concurrently? (2) What similarities and differences exist between reasons for using (a) vaped nicotine vs. vaped cannabis, and (b) vaped cannabis vs. smoked cannabis? (3) Are reasons for individual and concurrent use of nicotine and cannabis associated with product use behaviors?

## Methods

Data are from an anonymous, cross-sectional pilot survey of *n* = 112 co-users of vaped nicotine and cannabis recruited online from June 2020-August 2020 using CloudResearch, a third-party platform that interfaces with Amazon Mechanical Turk (mTurk) (Litman et al., 2017). The survey was promoted on mTurk using a Human Intelligence Task, which provided interested mTurk workers the opportunity to complete a short informed consent and eligibility screening (via Research Electronic Database Capture (REDCap) that assessed their past month use of alcohol, tobacco, and other substances (Harris et al., 2019). The screener took approximately 1 min to complete, and assessed individuals’ age, country/state of residence, and past month use of (1) alcohol, (2) tobacco (i.e., cigarettes, cigars, smokeless tobacco), (3) e-cigarettes (with nicotine, with only flavoring), (4) cannabis (assessed separately as smoked, vaped, edible, oral sprays/tinctures/capsules, and CBD-only products), and (5) other illicit drugs (including cocaine/crack, opiates, psychedelics, and club drugs). Those eligible for the main survey were (1) aged 18 years or older; (2) residents of the country of Canada, or a U.S. state with an active adult-use or medical cannabis policy in place; (3) past 30-day users of nicotine-containing e-cigarette or e-cigarettes that contain only flavoring; (4) past 30-day users of vaped cannabis; (5) those who usually used their vaping products at least monthly; and (6) those who had an mTurk Human Intelligence Task approval rating of 80% or higher, which is a marker of the quality of submissions completed by mTurk workers (Mellis & Bickel, 2020).

Eligible individuals were redirected to the main survey and asked to check a box to provide their consent to proceed with the full study. The main survey assessed nicotine and cannabis use behaviors for inhaled modes of administration, and took approximately 25-30 min to complete, depending on participants’ use of different inhaled nicotine and cannabis products. Participants who completed the main survey were paid $1.75, and those who passed all three attention check questions (e.g., “Select the color blue from the list.”) were paid an additional $3.25, for a total possible compensation of $5.00. In total, 2641 mTurk workers completed the eligibility screening. Among them, 5.6% (*n* = 148) were eligible to complete the main study. Among eligible participants, 121 completed the survey. After reviewing the data for response quality, duplicate responses by identification number and responses to attention check questions, 112 cases were retained in the final data set. Eighty-two percent of those who were eligible completed the main survey, while 76% of those who were eligible completed the survey and provided valid data (The American Association for Public Opinion Research (AAPOR), 2016). Methods for this project were reviewed and approved by the Institutional Review Board at the State University of New York at Buffalo (protocol #00003882).

### Measures

#### Reasons for using measures vaped cannabis, vaped nicotine, and smoked cannabis

All participants responded to parallel sets of measures to assess reasons for using vaped cannabis, vaped nicotine, and smoked cannabis. Selected items were adapted from studies in the published literature related to reasons for vaping cannabis or nicotine, as well as validated measures from national-level surveys (Lee et al., 2016; Aston et al., 2019; Berg et al., 2018; Etter, 2015; Hyland et al., 2017; McDonald et al., 2016; Popova et al., 2017; Shiplo et al., 2016). Participants indicated their agreement with each item as it related to their use of each product using a six-point scale ranging from 0 (not at all true) to 5 (very true). Item phrasing was adapted to suit the specific behavior being assessed, e.g., “The level of high I feel” for cannabis use, “The level of buzz I feel” for nicotine use. Table 1 provides an overview of items administered to participants.

**Table 1:** List of individual survey items used to construct reason for use subscales

	Cronbach’s alpha			
	Cannabis vaping	Nicotine vaping	Cannabis smoking	Co-use^b^
**User experience**				
The level of effect I feel	0.84	0.78	0.86	---
How satisfied it makes me feel				
How long I feel the effect after use				
The control I have over how much I use				
The time it takes for me to feel the effect				
I like it				
**Product/substance**				
The price	0.67	0.78	0.78	---
The taste				
The purity of the product				
The variety of product that is available to me				
The amount I need to feel the effect				
**Utility**				
I can vape in places where I cannot smoke^a^	0.67	0.79	0.66	---
It is convenient				
How easy it is to do				
I feel less judged by others about my use				
**Harm reduction**				
It might be less harmful to other people around me	0.85	0.89	0.94	---
It might be less harmful to my health				
I think vaping will help me to quit smoking^a^				
I think vaping will help me to cut back on my smoking^a^				
**Instrumentality**				
Using cannabis increases the buzz I get from nicotine	---	---	---	0.78
Using nicotine increases the buzz I get from cannabis				
I use nicotine when I cannot use cannabis				
**Displacement**				
I use cannabis when I cannot use nicotine	---	---	---	0.76
I have tried to reduce my use of nicotine by replacing it with cannabis				
I have tried to reduce my use of cannabis by replacing it with nicotine				
**Social context**				
I use cannabis or nicotine in different places (home, school, work, bars, parties)	---	---	---	0.78
I use cannabis or nicotine around different people (friends, peers, family)				
**Experimentation**				
I like to experiment with different products but do not use any regularly	---	---	---	0.59
I do not use cannabis or nicotine in any sort of sequence				
The use of one product had nothing to do with use of the other product				

^a^Items asked for cannabis and nicotine vaping only

^b^Measures of internal consistency from Berg et al. (2018): instrumentality, 0.81; displacement, 0.72; social context, 0.80; experimentation, 0.55

The list of items was reviewed by two independent raters with expertise on cannabis and/or tobacco use (D.S. and R.L.C.), and were qualitatively classified into four domains representing reasons for use across each product: (1) *user experience*; (2) *product or substance*; (3) *product utility*; and (4) *harm reduction*. Correlations between individual items were assessed to determine their suitability for inclusion within each subscale, and questionable items were brought up for further discussion between raters. The final subscale classifications can be viewed in Table 1. Individual item means, standard deviations, and inter-item correlations for cannabis vaping, nicotine vaping, and cannabis smoking can be viewed in Supplemental files 1-3. Analyses demonstrated good internal consistency among items included in each subscale (Cronbach’s alphas range, 0.66 (utility of cannabis smoking) to 0.94 (harm reduction issues related to cannabis smoking).

#### Reasons for nicotine and cannabis co-use

All participants responded to a series of 11 items previously published by Berg et al. (2018) designed to assess reasons for tobacco and cannabis co-use (Berg et al., 2018). Item phrasing was altered to reflect use of nicotine products instead of tobacco products, because “nicotine” encompasses the use of vaping products as well as tobacco cigarettes. Participants were asked to rate their level of agreement with each item using a scale ranging from 0 (not at all true) to 6 (extremely true). Each of the 11 items was classified into one of four subscales based on previous work: (1) Instrumentality (e.g., “Using cannabis increases the buzz I get from nicotine”); (2) displacement (e.g., “I’ve tried to reduce my use of nicotine by replacing it with cannabis”); (3) social context (e.g., “I use cannabis or nicotine in different places (home, school, work, bars, parties)”); and, (4) experimentation (e.g., “I don’t use cannabis or nicotine in any sort of sequence”). The classification of each of the items can be viewed in Table 1, while individual item means, standard deviations, and inter-item correlations can be viewed in Supplemental Table 4. Cronbach’s alpha ratings for each subscale ranged from 0.59 (experimentation) to 0.78 (instrumentality, social context). Measures of internal consistency for these subscales were similar in this sample to previously reported estimates (range, 0.55 (experimentation) to 0.81 (instrumentality)) (Berg et al., 2018). Supplemental Table 5 outlines associations between the four subscales for individual product use (for cannabis vaping, nicotine vaping, and cannabis smoking, respectively) and reasons for nicotine-cannabis co-use.

#### Frequency of monthly nicotine and cannabis use

The Daily Drinking Questionnaire was adapted to assess patterns of product use (Collins et al., 1985). For each product (vaped cannabis, vaped nicotine, smoked cannabis, or tobacco cigarettes), participants completed a 7-day Time Line Follow Back (TLFB) asking about the average number of use sessions on each day of a typical use week. Participants who smoked tobacco cigarettes were asked to report the number of cigarettes smoked each day on the days where smoking took place. Monthly use was calculated by taking the number of use days reported on the TLFB and multiplying it times four; the range of use days in the past month was 0 (no use)-28 (daily use). Cigarette use was classified any vs. no use due to the distribution of cigarette smoking within the sample. The TLFB was also used to estimate the number of monthly use sessions by summing the values of reported daily use sessions across the week, which were multiplied by four to generate a proxy measure of total monthly use sessions.

#### Co-use behaviors

Individual items were administered to assess frequency of chasing behaviors (four items) and co-administration practices (four items) (Tucker et al., 2019). Items assessing chasing behaviors assessed different ordering of product use, and asked participants to rate how often they engaged in each practice (response options: all the time, sometimes, rarely, never). Similarly, participants were asked to report the frequency with which they engaged in one of four co-administration practices: (1) mixing nicotine and cannabis oil together for use in a vaping device; (2) mixing tobacco and cannabis together for use in a dry herb vaporizer; (3) mixing tobacco and cannabis together in a joint, blunt, bowl bong, or other smoking device; and (4) smoking cigarettes dipped in hash oil. For each practice, the response options were never, once in my life, 1-10 times in my life, 11-19 times in my life, over 20 times in my life. Responses for all items assessing chasing and co-administration behaviors were recoded to reflect ever versus never engaging in each co-use practice.

#### Sociodemographic measures

Participants reported their age, gender, race, ethnicity, level of education, total annual household income, and country/state of residence. The cannabis policy environment in which participants’ resided (adult-use permitted vs. medical use only) was derived using the state or country of residence at the time of the survey. A subset of the Global Assessment of Individual Needs Short Screener (GAIN-SS) was administered to all participants to assess individuals’ degree of past-year internalizing, externalizing, and substance use problem behaviors (Dennis et al., 2006). Items were scored according to published specifications, with scores of 0 indicating an individual is unlikely to exhibit problems requiring clinical intervention, and scores of 5 indicating an individual is very likely to exhibit problems requiring clinical intervention.

### Statistical analyses

Ratings for each reason for use subscale were treated as interval data. Means and standard deviations (SD) were calculated to examine average ratings for each reason for use subscale across participants. Paired *t* tests were used to determine differences between individual reason for use subscale ratings according to type of product, with a focus on comparing modes (i.e., vaped cannabis to vaped nicotine) and substance (i.e., vaped cannabis to smoked cannabis). A series of regression models were constructed to assess associations between reasons for individual product use (user experience, product/substance, utility, and harm reduction for nicotine vaping, cannabis vaping, and cannabis smoking, respectively) and reasons for co-use (instrumentality, displacement, social context, and experimentation) on monthly substance use frequency. Linear regression analyses were performed to examine associations between reasons for product use and monthly use sessions. Monthly use sessions were chosen as the outcome variable representing product use due to superior model fit indices over monthly use days and monthly quantity-frequency of use. Each of these outcomes was right-skewed and was transformed using the natural log to approximate a normal distribution more readily. Logistic regression modeling was used to examine associations between reasons for product use and odds of ever engaging in co-use behaviors, including chasing and co-administration. All models adjusted for age (continuous), gender, GAIN-SS subscale scores (internalizing, externalizing, and substance use problems, all entered as continuous variables), cigarette smoking status, and the number of monthly use sessions for the other concurrently used substances. Analyses were conducted using Stata version 16.0, and *p* values < 0.05 were statistically significant.

## Results

### Demographics

Participant demographic characteristics can be viewed in Table 2. On average, participants were age 30 (SD ± 8.4 years), most (63.4%) were male and identified as being White, non-Hispanic (72.3%). The sample was relatively evenly distributed across income and educational strata. Most participants resided within areas with adult-use cannabis policies in place (64.3%). Sample members generally expressed a high degree of past year internalizing symptoms (65.2%), a moderate degree of past year externalizing symptoms (61.6%), and moderate degree of past year substance use problems (75.9%).

**Table 2:** Sociodemographic characteristics of 112 nicotine and cannabis users

		*n*	Estimate (mean ± SD or %)
Age (mean ± SD), in years		112	30 ± 8.4
Sex	Male	71	63.4
	Female	38	33.9
	Other	3	2.7
Race/ethnicity	White, non-Hispanic	81	72.3
	All other races	31	27.7
Income	< $25,000	19	17.0
	$25,001-$50,000	29	25.9
	$50,001-$75,000	23	20.5
	$75,001-$100,000	20	17.9
	Over $100,000	19	17.0
	I prefer not to answer	2	1.8
Education	High school or equivalent	14	12.5
	Some college	34	30.4
	Associates	12	10.7
	Bachelors	41	36.6
	Graduate	11	9.8
Cannabis policy environment	Medical only	40	35.7
	Adult use	72	64.3
Survey mode	Computer/laptop	101	90.1
	Tablet	1	0.9
	Mobile phone	10	8.9
GAIN-SS internalizing (mean ± SD)		112	3.0 ± 1.6
GAIN-SS internalizing (by category)	Low	0	0
	Moderate	39	34.8
	High	73	65.2
GAIN-SS externalizing (mean ± SD)		112	1.8 ± 1.5
GAIN-SS externalizing (by category)	Low	0	0
	Moderate	69	61.6
	High	43	38.4
GAIN-SS substance use problems (mean ± SD)		112	2.0 ± 1.5
GAIN-SS substance use problems (by category)	Low	0	0
	Moderate	85	75.9
	High	27	24.1

Estimates reflect percentages except where noted, percentages are rounded and therefore may not total to 100%

*SD* standard deviation, *GAIN-SS* Global Appraisal of Needs Short Screener (Dennis et al. 2003), Low = 0, Moderate = 1-2, High = 3-5; score reflects likelihood of needing clinical services to address internalizing, externalizing, and/or substance use problems

### Reasons for individual product use and co-use

**Fig. 1:**
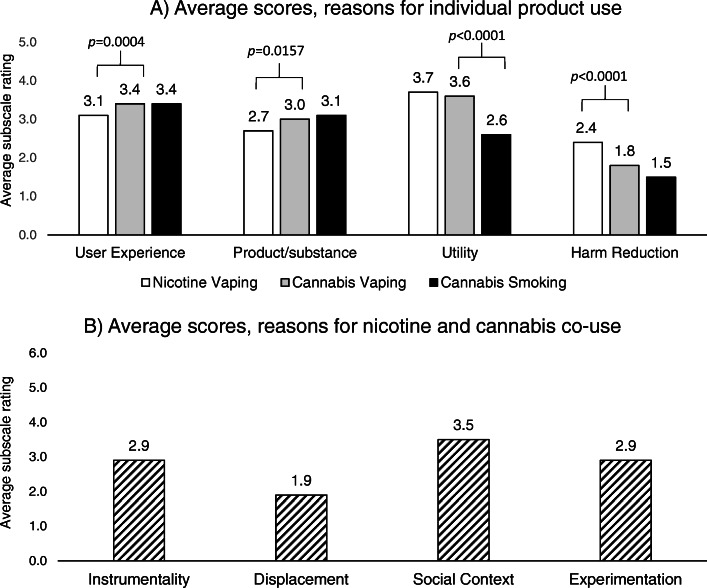
Average subscale ratings for (**a**) individual product use and (**b**) reasons for nicotine/cannabis co-use (*n* = 112). Scales range from 0 (not at all)-5 (very much) for cannabis vaping, nicotine vaping, and cannabis smoking reasons for use subscales; scale ranges from 0 (not at all)-6 (very much) for co-use subscales. Mean represents the pooled average score across all participants

Mean subscale ratings for using vaped nicotine, vaped cannabis, and smoked cannabis are presented in Fig. 1a. Both nicotine vaping and cannabis vaping received highest overall endorsement for utility-related reasons for use (nicotine vaping, mean = 3.7 (SD = 1.1); cannabis vaping, mean = 3.6 (SD = 1.0). Evaluations of mean differences suggested that reasons for product use related to the user experience and the product/substance being consumed were more similar for cannabis vaping and cannabis smoking, while reasons for use related to the utility of products were more similar for cannabis vaping and nicotine vaping. There were small, statistically significant differences in mean scores for user experience-related reasons for use between cannabis vaping and nicotine vaping (mean (SD), 3.4 (± 1.0) vs. 3.1 (± 1.1); mean difference = 0.37, *t* = 3.65, *p* = 0.0004), as well as product/substance-related reasons for use (mean (SD), 3.0 (± 1.0) vs. 2.7 (± 1.2); mean difference = 0.26, *t* = 2.45, *p* = 0.0157). Conversely, the average rating for utility-related reasons for use were significantly higher for cannabis vaping compared to cannabis smoking (mean (SD), 3.6 (± 1.0) vs. 2.6 (± 1.2); mean difference = 0.98, *t* = 7.84, *p* < 0.0001). Harm reduction-related reasons for product use exhibited larger differences, with cannabis vaping receiving significantly lower average ratings compared to nicotine vaping (mean (SD), 1.8 (± 1.4) vs. 2.4 (± 1.6), mean difference = −0.65, *t* = −4.24, *p* < 0.0001). Mean ratings for each reason for co-use subscale score can be viewed in Fig. 1b. Participants provided the strongest endorsements for social context-related factors as reasons for co-use (mean (SD), 3.5 (± 1.8)), followed by instrumentality (mean (SD), 2.9 (± 1.8), experimentation (mean (SD), 2.9 (± 1.5)), and displacement (mean (SD), 1.9 (± 1.6)).

### Associations between reasons for product use, co-use, and use behaviors

Table 3 displays the association between reasons for individual product use and co-use and monthly substance use sessions after controlling for other factors. After accounting for age, gender, psychosocial correlates of substance use, and concurrent use of other nicotine and cannabis products, increases in ratings for utility-related (*b* = 0.32; 95% CI, 0.03-0.60) and harm reduction-related (*b* = 0.21; 95% CI, 0.04-0.37) reasons for nicotine vaping were significantly associated with greater frequency of monthly nicotine vaping use sessions. Interestingly, increases in ratings for utility-related factors related to cannabis smoking were associated with decreasing monthly frequency of nicotine vaping use sessions (*b* = −0.41; 95% CI, −0.72-−0.10). Increasing endorsement of product/substance-related reasons for cannabis smoking were positively associated with increases in monthly cannabis smoking use sessions (*b* = 0.33; 95% CI, 0.03-0.64). No associations between reasons for individual product use and monthly cannabis vaping use session were detected. Individual product reasons for use also corresponded with increased odds of ever engaging in chasing behaviors, such that user-experience-related reasons for cannabis vaping were associated with a twofold increase in odds of ever having engaged in chasing, and utility-related reasons for cannabis smoking were associated with nearly threefold greater odds of ever having engaged in chasing.

**Table 3:** Linear regressions examining the association between reasons for individual product use and co-use with monthly use sessions for (a) nicotine vaping use, (b) cannabis vaping, and (c) cannabis smoking (*n* = 112)

	Monthly use sessions											
	(A) Nicotine vaping				(B) Cannabis vaping				(C) Cannabis smoking			
	*b*	95% L	95% U	*p* value	*b*	95% L	95% U	*p* value	*b*	95% L	95% U	*p* value
Nicotine vaping												
User experience	0.24	−0.19	0.67	0.28	0.37	−0.00	0.74	0.05	−0.12	−0.47	0.24	0.51
Product/substance	−0.12	−0.51	0.27	0.53	−0.16	−0.50	0.18	0.35	0.11	−0.21	0.43	0.50
Utility	0.32*	**0.03**	**0.60**	**0.03**	−0.17	−0.43	0.08	0.18	−0.17	−0.40	0.07	0.16
Harm reduction	0.21*	**0.04**	**0.37**	**0.01**	−0.06	−0.21	0.09	0.44	0.05	−0.09	0.19	0.46
Cannabis vaping												
User experience	−0.05	−0.45	0.35	0.81	0.14	−0.16	0.45	0.35	−0.05	−0.35	0.24	0.73
Product/substance	0.04	−0.41	0.50	0.85	0.20	−0.15	0.55	0.25	−0.12	−0.45	0.22	0.50
Utility	0.19	−0.10	0.47	0.20	−0.02	−0.24	0.20	0.88	0.12	−0.10	0.33	0.28
Harm reduction	−0.16	−0.38	0.06	0.14	0.08	−0.09	0.25	0.35	0.11	−0.05	0.27	0.18
Cannabis smoking												
User experience	−0.05	−0.45	0.35	0.81	0.26	−0.07	0.58	0.12	−0.17	−0.45	0.11	0.24
Product/substance	0.38	−0.05	0.81	0.08	−0.25	−0.61	0.10	0.16	0.33*	**0.03**	**0.64**	**0.03**
Utility	−0.41*	−**0.72**	−**0.10**	**0.01**	−0.04	−0.31	0.23	0.75	0.19	−0.04	0.42	0.10
Harm reduction	−0.04	−0.26	0.17	0.68	0.08	−0.10	0.26	0.37	−0.02	−0.18	0.13	0.78
Reasons for co-use												
Instrumentality	0.26**	**0.08**	**0.44**	**0.01**	0.04	−0.11	0.20	0.58	−0.06	−0.20	0.08	0.37
Displacement	−0.20	−0.40	0.01	0.06	0.07	−0.10	0.24	0.43	0.14	−0.01	0.29	0.07
Social context	0.02	−0.14	0.17	0.83	0.01	−0.12	0.14	0.90	0.05	−0.06	0.17	0.35
Experimentation	−0.01	−0.19	0.18	0.95	−0.06	−0.21	0.09	0.45	0.06	−0.07	0.20	0.35

Bold and starred values indicate statistically significant effects. All models adjusted for age, gender, GAIN-SS subscale scores (internalizing, externalizing, and substance use problems), cigarette smoking status, and monthly product use sessions

After adjustment, only increased endorsement of instrumentality-related reasons for co-use corresponded with increasing monthly nicotine vaping use sessions (*b* = 0.26; 95% CI, 0.08-0.44). Greater endorsement of instrumentality-related reasons for co-use corresponded with a threefold increase in odds of ever chasing (aOR, 3.06; 95% CI, 1.29-7.30). Greater endorsement of experimentation-related reasons for co-use was inversely associated with odds of ever chasing (aOR, 0.47; 95% CI, 0.24-0.91). There were no associations between individual product reasons for use, or reasons for co-use, on odds of ever having engaged in nicotine-cannabis co-administration (Table 4).

**Table 4:** Logistic regressions examining the association between reasons for individual product use and co-use with odds of lifetime engagement in (a) sequential use practices (“chasing”), and (b) co-administration behaviors (*n* = 112)

	(A) Chasing				(B) Co-administration			
	aOR	95% L	95% U	*p* value	aOR	95% L	95% U	*p* value
Nicotine vaping								
User experience	0.86	0.24	3.02	0.81	1.06	0.47	2.39	0.88
Product/substance	1.61	0.55	4.68	0.38	1.21	0.59	2.49	0.60
Utility	1.22	0.50	3.00	0.66	0.90	0.52	1.56	0.72
Harm reduction	1.08	0.64	1.80	0.78	1.10	0.80	1.52	0.57
Cannabis vaping								
User experience	2.18*	**1.01**	**4.71**	**0.05**	0.87	0.44	1.73	0.69
Product/substance	1.34	0.66	2.70	0.41	1.64	0.74	3.67	0.23
Utility	0.44	0.18	1.05	0.07	0.89	0.55	1.44	0.65
Harm reduction	1.18	0.66	2.11	0.58	1.17	0.78	1.74	0.45
Cannabis smoking								
User experience	1.00	0.40	2.53	0.99	0.82	0.41	1.61	0.56
Product/substance	0.33	0.08	1.32	0.12	1.01	0.47	2.16	0.98
Utility	2.99*	**1.07**	**8.35**	**0.04**	1.55	0.85	2.83	0.15
Harm reduction	0.95	0.52	1.72	0.87	1.05	0.71	1.56	0.82
Reasons for co-use								
Instrumentality	3.06*	**1.29**	**7.30**	**0.01**	1.27	0.90	1.81	0.17
Displacement	0.82	0.34	1.93	0.64	1.41	0.93	2.13	0.10
Social context	0.85	0.49	1.47	0.56	1.02	0.77	1.34	0.89
Experimentation	0.47*	**0.24**	**0.91**	**0.02**	1.05	0.74	1.51	0.78

Bold and starred values indicate statistically significant effects. All models adjusted for age, gender, GAIN-SS subscale scores (internalizing, externalizing, and substance use problems), cigarette smoking status, and monthly product use sessions

## Discussion

Our pilot study is the first to directly compare reasons for using nicotine and cannabis vaping products within a sample of U.S. adult vapers. Findings show that reasons for individual use of nicotine and cannabis vaping products were rated most favorably for utility-related factors, while reasons for co-use were most strongly endorsed for social context-related reasons. Results also indicated that there were some differences in participant ratings representing reasons for using vaped nicotine and smoked cannabis when compared to vaped cannabis, and reasons for individual product use and co-use exhibited significant associations with increasing monthly use of these products. These data can serve as a starting point for improving understanding of the similarities and differences between nicotine and cannabis vaping, and the importance of considering differences by both substance and mode when examining the co-use of these products.

Compared to cannabis vaping, reasons for vaping nicotine elicited lower overall ratings for user experience and product/substance, and higher overall ratings related to harm reduction. Alternatively, compared to cannabis vaping, reasons for smoking cannabis were statistically similar for user experience and product/substance-related factors, and utility-related reason for use ratings were significantly lower. Utility-related reasons for use were similar for nicotine vaping and cannabis vaping and were associated with increasing monthly use of nicotine. Taken together, this would suggest that vaping as a mode of drug delivery serves a purposeful application that differs by substance, with nicotine vaping being more closely related to reducing tobacco smoking-related harms, and cannabis vaping being more closely related to circumventing social problems commonly posed by cannabis smoking. These observations are consistent with studies examining each product individually (Lee et al., 2016; Saddleson et al., 2016; Aston et al., 2019; Popova et al., 2017; Pokhrel et al., 2015), and is likely reflecting broader differences in the legality and risk perceptions related to the substances themselves. Smoked tobacco is legal and is accurately perceived as harmful to health, while cannabis remains illegal in many areas and is increasingly perceived as less risky or health promoting due to increasing medical and therapeutic use (Azofeifa et al., 2016; Hall & Kozlowski, 2018; National Academies of Science Engineering and Medicine, 2017).

While social context was the most highly rated reason for co-use of nicotine and cannabis, instrumentality also consistently emerged as a significant reason for co-use. After controlling for other factors, we observed that instrumentality-related reasons for use were significantly associated with sequential use of these substances. While chasing nicotine with cannabis may occur in a variety of social settings, results suggest that enhancement of the intoxicating effects of cannabis through co-use of nicotine products is the main reason why adults co-use nicotine and cannabis. Results suggesting that monthly nicotine vaping sessions increase alongside greater endorsement of instrumentality-related reasons for co-use provide added support for this concept. In line with the literature on sequential use practices (Ream et al., 2008; Peters et al., 2020), this may lead to increased dependence on nicotine and contribute to reinforcing effects of these substances and promote continued or escalating use. Similarly, utility-related and harm reduction-related reasons for nicotine vaping also exerted a significant, positive association with increasing monthly use. In aligning these findings, this suggests aspects of nicotine vaping believed to be beneficial (utility and harm reduction) may occur alongside reasons for cannabis use associated with greater nicotine dependence (instrumentality). This points toward the importance of conducting future studies on sequential use practices and how they may contribute to nicotine dependence among vapers. As cannabis use and nicotine vaping become more prevalent, monitoring how patterns of sequential use and co-administration may shift alongside these trends will be an important direction for work aiming to monitor the health effects arising from nicotine vaping and cannabis use, respectively.

Once we adjusted for other factors, we did not find support that co-use was associated with substituting nicotine and cannabis products (displacement), or social context factors related to co-use. Experimentation-related reasons for use were inversely associated with history of engaging in chasing behaviors. Given that Berg et al. conducted their initial study on reasons for co-use among young adults, this suggests at least two things. First, the reasons for nicotine and cannabis co-use are different, or shift, as age increases and as use of nicotine and cannabis progresses. Second, there may be different reasons for co-use among older adults that remain unaccounted for in this scale. Future research on reasons for co-use among adults should explore this issue.

### Strengths and limitations

This pilot study provides preliminary data examining the association between reasons for product use and co-use behaviors, including direct comparisons of reasons for using vaped nicotine, vaped cannabis, and smoked cannabis. While the measures included in this study provide a more detailed assessment of these issues than what is available in larger-scale surveys (Geissler et al., 2020), there are some limitations. First, these findings should be considered exploratory considering the small sample size and convenience sampling method. While mTurk is a useful resource for exploratory studies of substance use and characterizing relationships between measured behavioral constructs, this population tends to skew younger, is less fully employed, and has a disproportionately larger number of substance users than the general population, which likely limits the overall generalizability of our results (Mellis & Bickel, 2020). Further, the sample size limited our ability to utilize multivariate statistical techniques to classify data on reasons for individual product use and co-use, or formally reexamine subscale classifications related to nicotine and cannabis co-use. However, the associations observed within our data on reasons for use and use behaviors align with other published studies on this topic (Lee et al., 2016; Saddleson et al., 2016; Berg et al., 2018; Popova et al., 2017; Pokhrel et al., 2015), giving credibility to our findings in light of this limitation. Additionally, the list of items included in this study outlining reasons for product use was derived from the published literature and national surveys. While this gave us a sound pool of items to draw upon, it is possible that other reasons for co-use exist among concurrent vapers. Future studies, including qualitative research, should be conducted to provide more robust assessments of reason for nicotine and cannabis use in vaping devices in larger, more robust samples. The cross-sectional nature of this study makes it impossible to determine temporality between reasons for individual product use, co-use, and related use frequency and behaviors. Finally, our study did not include other subsets of co-users (e.g., exclusive e-cigarette users that only smoke cannabis), which limited our ability to compare differences in reasons for use that may exist among those who use product differently than the current sample. Future research may expand on this project to include suitable comparisons to garner a more robust understanding of reasons for using nicotine and cannabis vaping products, and their linkages with co-use practices and use behaviors, including those beyond lifetime co-use practices.

## Conclusions

Our findings suggest that vaping as a mode of drug delivery serves a purposeful application that differs by substance, with nicotine vaping being more closely related to reducing tobacco smoking-related harms, and cannabis vaping being more closely related to circumventing social problems commonly posed by cannabis smoking. Lifetime engagement in sequential co-use practices and more frequent monthly nicotine vaping were associated with reasons for use that related to enhancing the intoxicating effects of cannabis. Findings provide a basis for more detailed examinations of reasons for nicotine and cannabis product use, and their co-use, among adult populations who use vaping products.

## Supplementary Information

**Additional file 1:** Supplemental Table 1. Means, Standard Deviations, and Inter-Item Correlations, Reasons for Cannabis Vaping (*n*=112). Supplemental Table 2. Means, Standard Deviations, and Inter-Item Correlations, Reasons for Nicotine Vaping (*n*=112). Supplemental Table 3. Means, Standard Deviations, and Inter-Item Correlations, Reasons for Cannabis Smoking (*n*=112). Supplemental Table 4. Means, Standard Deviations, and Inter-Item Correlations, Reasons for Co-use (*n*=112). Supplemental Table 5. Correlations between subscales outlining reasons for individual product use and co-use (*n*=112)

## Data Availability

The datasets used and/or analyzed during the current study are available from the corresponding author on reasonable request.
